# Gender, Obesity and Repeated Elevation of C-Reactive Protein: Data from the CARDIA Cohort

**DOI:** 10.1371/journal.pone.0036062

**Published:** 2012-04-30

**Authors:** Shinya Ishii, Arun S. Karlamangla, Marcos Bote, Michael R. Irwin, David R. Jacobs, Hyong Jin Cho, Teresa E. Seeman

**Affiliations:** 1 Department of Geriatric Medicine, University of Tokyo, Bunkyo-ku, Tokyo, Japan; 2 Division of Geriatrics, Department of Medicine, University of California Los Angeles, Los Angeles, California, United States of America; 3 Department of Sociology and Social Policy, University of Murcia, Murcia, Spain; 4 Cousins Center for Psychoneuroimmunology, Semel Institute for Neuroscience and Human Behavior, University of California Los Angeles, Los Angeles, California, United States of America; 5 Division of Epidemiology and Community Health, School of Public Health, University of Minnesota, Minneapolis, Minnesota, United States of America; Innsbruck Medical University, Austria

## Abstract

C-reactive Protein (CRP) measurements above 10 mg/L have been conventionally treated as acute inflammation and excluded from epidemiologic studies of chronic inflammation. However, recent evidence suggest that such CRP elevations can be seen even with chronic inflammation. The authors assessed 3,300 participants in The Coronary Artery Risk Development in Young Adults study, who had two or more CRP measurements between 1992/3 and 2005/6 to a) investigate characteristics associated with repeated CRP elevation above 10 mg/L; b) identify subgroups at high risk of repeated elevation; and c) investigate the effect of different CRP thresholds on the probability of an elevation being one-time rather than repeated. 225 participants (6.8%) had one-time and 103 (3.1%) had repeated CRP elevation above 10 mg/L. Repeated elevation was associated with obesity, female gender, low income, and sex hormone use. The probability of an elevation above 10 mg/L being one-time rather than repeated was lowest (51%) in women with body mass index above 31 kg/m^2^, compared to 82% in others. These findings suggest that CRP elevations above 10 mg/L in obese women are likely to be from chronic rather than acute inflammation, and that CRP thresholds above 10 mg/L may be warranted to distinguish acute from chronic inflammation in obese women.

## Introduction

With growing evidence linking chronic inflammation to various major disease processes, interest in C-Reactive Protein (CRP) - a robust marker of systemic inflammation – has increased sharply. Efforts to elucidate the role of CRP in cardiovascular and other disease processes have necessitated consideration of how to interpret very high levels of CRP, specifically whether values above a certain level (e.g., 10 mg/L) reflect acute inflammation, such as due to recent or ongoing infection, and should therefore not be used as a marker of chronic inflammation. Clinical guidelines recommend repeat CRP measurement (after at least 2 weeks) in those with an initial reading >10 mg/L, to distinguish between short-term and more sustained elevation [1]–[3]. In research studies, the current convention is to exclude those with CRP >10 mg/L [3]. However, there are multiple reasons to question the 10 mg/L threshold for distinguishing acute from chronic inflammation. First, CRP values greater than 10 mg/L are not uncommon; multiple studies indicate that 5% or more of the population has such values [4]–[7]. Second, some data suggest that even a one-time CRP value >10 mg/L does have long-term health implications. Indeed, individuals with CRP >10 mg/L had the highest risk of future vascular events in several cohorts, including healthy middle-aged women [4], middle-aged men and women [8], older men and women [9], and post-stroke patients [10]. Thus, CRP values >10 mg/L might indicate chronic inflammation in a large proportion of individuals, and this may especially be so in people with characteristics such as obesity and smoking, which are known risk factors for chronic inflammation.

Accordingly, our objective was to determine what proportion of patients, among those who do not report being sick recently, have CRP >10 mg/L; whether this proportion varies by patient characteristics; whether these patients actually show evidence of sustained CRP elevation over several years; and if a higher threshold could be used to improve the identification of those with one-time elevation. The latter, if found, would have important implications for the current practice of treating CRP values over 10 mg/L as indicative of acute infection and excluding them from analyses. To the extent that we find that many CRP values over 10 mg/L are, in fact, more likely indicative of chronically high levels, exclusion of such values from analyses should be re-considered.

## Methods

### Study Design and Population

The Coronary Artery Risk Development in Young Adults (CARDIA) is a bi-ethnic, prospective, multi-center study of the evolution of cardiovascular risk beginning in young adulthood. In 1985–1986, 5,115 participants, aged 18 to 30 years, were recruited at Birmingham, AL; Chicago, IL; Minneapolis, MN; and Oakland, CA, to achieve a balance at each site by race (black, white), gender, education (high school degree or less, more than high school), and age (18–24 years, 25–30 years [11]). Follow-up examinations occurred during 1987–1988 (Year 2), 1990–1991 (Year 5), 1992–1993 (Year 7), 1995–1996 (Year 10), 2000–2001 (Year 15), and 2005–2006 (Year 20). A majority of the surviving group has been examined at each follow-up (91%, 86%, 81%, 79%, 74%, and 72%, respectively). Before exclusions (detailed below), 4,086 participants who were examined at year 7 were eligible for this analysis. Serum CRP measurements were made in Years 7, 15, and 20. CRP values of those reporting having been sick during the 24 hours preceding their blood draw were coded as missing for that year – 8.8% (n = 355) for Year 7, 7.9% (n = 266) for Year 15 and 5.6% (n = 179) for Year 20, for a total of 800 excluded CRP measurements. In total, 167 participants were completely excluded from the analysis because they reported having been sick at one or more than one visits. Inclusion in the present analyses required availability of valid CRP data (when not sick in the previous 24 hours) on at least two of the three visits in years 7, 15 and 20 (n = 3,354). Those who were pregnant (n = 50) or had self-reported coronary artery disease (n = 4) at Year 7 were excluded, leaving a sample size of 3,300. The study was approved by an institutional review board at each CARDIA study site (Birmingham, AL; Chicago, IL; Minneapolis, MN; and Oakland, CA). All participants provided written informed consent at each examination. Detailed study design and cohort descriptions are available [12].

### Measurements: C-Reactive Protein

Plasma CRP assays for all samples were done using the BNII nephelometer from Dade Behring utilizing a particle enhanced immunonepholometric assay; the assay range is 0.175-1,100 mg/L, intra-assay coefficients of variation 2.3–4.4% and inter-assay coefficients of variation 2.1–5.7%. Technical error (including handling, storage, and assay variation) in blinded duplicate plasma aliquots at years 15 and 20 was 18.5% and 20.4%, respectively.

We defined one-time CRP elevation as having CRP >10 mg/L at exactly one visit, and repeated elevation as having CRP >10 mg/L at two or more visits.

### Measurements: Predictors of CRP Elevation

The predictors considered were participant characteristics at the initial CRP evaluation (Year 7), pre-specified based on literature review.

*Socio-demographic variables* examined were gender, race (African-American vs. Caucasian), pre-tax family income (Less than 25,000 dollars a year; 25,000 to 50,000; 50,000 to 75,000; and more than 75,000), and highest education grade completed (High School or Less, College Only, or Graduate School).

#### Health Behavior variables

Smoking status was coded as never-smoker, past-smoker, and current-smoker. A physical activity intensity score was computed by multiplying reported frequency of engagement in 13 exercise and recreation activities by the intensity of the activity [13]. Consumption of wine, beer, and liquor was assessed and summed to create an index of total alcohol consumption (ml/day). The total alcohol consumption was then categorized as non-drinker, moderate-drinker (0.1–12 ml/day) or severe-drinker (>12 ml/day).

#### Health status

Participants were asked about doctor-diagnosed high blood pressure, diabetes, high cholesterol, asthma, chronic obstructive pulmonary disease and cancer. Asthma and chronic obstructive pulmonary disease were combined into a binary variable, respiratory conditions. Information was also collected on medications for hypertension, aspirin, oral contraceptive pills and hormone replacement therapy. Use of oral contraceptive pills and hormone replacement therapy were combined into a binary variable, use of sex hormones. Body mass index (BMI) was calculated from measured height and weight.

### Analyses

Analyses were undertaken to answer the following three questions:

1. What patient characteristics are associated with CRP elevation above 10 mg/L one or more times, relative to no CRP elevation above 10 mg/L?
2. Of those with CRP elevated above 10 mg/L one or more times, are certain patient characteristics associated with greater likelihood of the CRP elevation being repeated rather than a one-time elevation?
3. Can we improve the identification of one-time CRP elevation by using different CRP thresholds in different groups?

First, the associations of socio-demographic, health status, and health behavior characteristics with the odds of CRP elevation above 10 mg/L one or more times (relative to no CRP elevation above 10 mg/L) were examined using multinomial logistic regression. Physical activity, BMI and age were included as continuous variables; the rest as categorical. In addition, we included a quadratic term for BMI (BMI was centered at the mean and then squared) and two gender by variable interactions (i.e., gender by BMI and BMI-squared) based on literature review [5], [7], [14]–[20].

Second, we examined the odds of repeated CRP elevation among those who had at least one episode of CRP elevation above 10 mg/L, as a function of the same demographic/health/lifestyle characteristics, using logistic regression. The model was made parsimonious using backward selection based on the Akaike Information Criterion [21]. A generalized Spearman correlation was used to identify the two most influential predictor variables in the logistic regression model [22]. Next, using the two most influential variables as predictors, regression tree analysis was performed to identify subgroups at high risk for CRP elevations being repeated rather than one-time elevations [23].

Finally, we examined the effects of various CRP thresholds on the probability of above-threshold elevations being one-time elevations in sub-groups defined by the two characteristics that were most influential in the logistic regression model.

Analyses used R statistical software version 2.9.0 (R Foundation, Vienna, Austria). All statistical tests were two-sided, and a *P*-value less than 0.05 was considered statistically significant.

## Results

The study sample (n = 3,300) was representative of the complete CARDIA Year 7 sample (n = 4,086) with respect to clinical characteristics at baseline. In the study sample, 2,972 (90.1%) had no episode of CRP elevation above 10 mg/L, while 225 (6.8%) had one-time elevation and 103 (3.1%) had elevation on at least two occasions (Table 1). Among the 3,300 participants included, 471 (14.3%) reported being sick at one visit, of whom 41 (8.7%) had CRP elevation at one or more non-sick visits. The prevalence of CRP elevation above 10 mg/L did not increase from year 7 (4.3%) to year 20 (3.7%). Of the 225 participants with one-time elevation above 10 mg/L, only 60 (1.8% of 3,300, 26.7% of 225) had no other CRP readings above 3 mg/L; the other 165 had at least one other reading above 3 mg/L, and 96 of them had at least one other reading above 6 mg/L, suggesting chronic rather than acute inflammation.

**Table 1:** Baseline (year 7) Characteristics of 3,300 Participants in the CARDIA Study, According to the C-reactive Protein (CRP) Categories* †.

Characteristics		No CRP elevation above10 mg/L	One-time CRP elevation	Repeated CRP elevation
		N = 2972	N = 225	N = 103
Age(years)		33(29,35)	33(30,35)	32(29,35)
Gender				
	male	47.6	29.3	11.7
	female	52.4	70.7	88.4
Race				
	Caucasian	56.0	40.4	24.3
	African-American	44.0	59.6	75.7
Income				
	<$25 K/year	30.4	37.7	54.4
	$25-$50 K/year	36.9	39.6	33.0
	$50-$75 K/year	18.0	14.1	11.7
	>$75 K/year	14.8	8.6	1.0
Education				
	high school	26.1	30.8	35.9
	college	54.1	52.9	55.3
	graduate school	19.9	16.3	8.7
Smoking status				
	current smoker	24.2	30.7	29.4
	former smoker	15.8	20.0	12.8
Physical activity (100 EU)		2.8(1.4,4.9)	2.1(1.1,4.3)	1.6(0.6,3.0)
Alcohol use‡				
	non-drinker	44.2	48.9	62.8
	<1 glass a day	28.2	24.9	17.7
	> = 1 glass a day	27.6	26.2	19.6
Body mass index (kg/m^2^)		25.1(22.4,28.6)	29.1(25.4,34.8)	35.8(30.8,41.5)
Comorbidities §				
	hypertension	9.0	12.4	24.3
	diabetes mellitus	3.1	4.9	4.9
	hyperlipidemia	11.8	11.3	12.9
	respiratory conditions ||	10.8	14.8	18.6
	cancer	1.9	2.7	0
Medication use				
	sex hormones ¶	22.5	28.3	24.2
	aspirin	3.5	3.6	1.9
	anti-hypertensives	1.4	2.2	4.9

Abbreviations; K, 1,000: EU, exercise units:

* Median values and interquartile ranges are shown for continuous variables and percentages for categorical variables.

† Percentages may not add up to 100 because of rounding.

‡ 1 drink/day approximately corresponds to 12 g alcohol/day.

§ Comorbidities were self-reported in response to the questions “Has a doctor or nurse ever told you that you have …”.

|| Asthma and/or chronic obstructive pulmonary disease.

¶ Use of oral contraceptive pills or menopausal hormone replacement therapy. The percentages given in this row are among women only.

**Figure 1:**
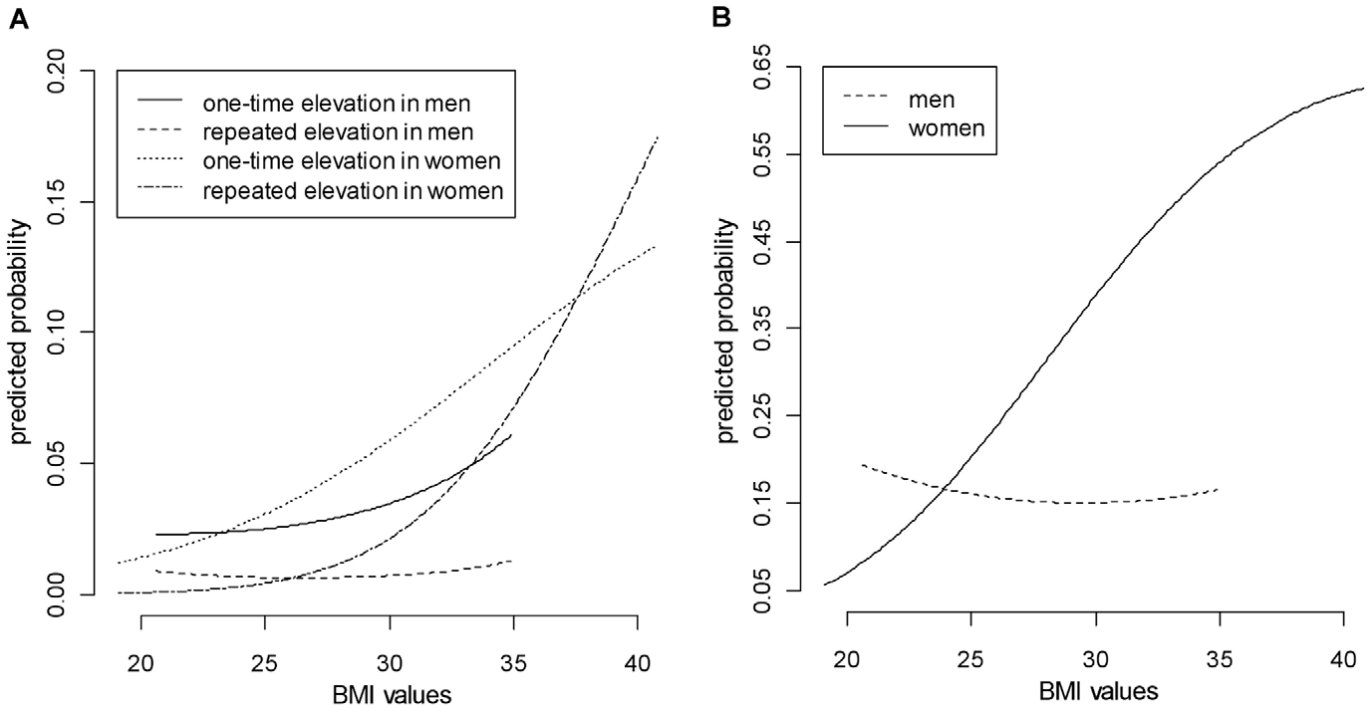
Predicted probabilities of C-Reactive Protein (CRP) elevation above 10 mg/L as function of body mass index (BMI). The predicted probabilities were computed from multiple logistic regression models while all other covariates were held constant (at reference values for categorical covariates and median values for continuous covariates). Panel A. Model-predicted probabilities of one-time and repeated CRP elevation. Panel B. Model-predicted probabilities of exactly one-time CRP elevation among those with one or more CRP elevations above 10 mg/dL.

Multinomial logistic regression of one-time and repeated CRP elevation (relative to no CRP elevation above 10 mg/L) demonstrated that higher income was associated with *decreased* odds of repeated CRP elevation, and both current and past smoking were associated with *increased* odds of one-time CRP elevation. Women were *more* likely than men to have both one-time and repeated CRP elevation, even after controlling for the use of sex hormones, which was also associated with *increased* odds of both one-time and repeated CRP elevation (Table 2). High BMI was associated with increased odds of both one-time and repeated CRP elevation, but the magnitude of the association was smaller in men than in women. In women, the odds of both one-time and repeated CRP elevation increased dramatically with BMI (Figure 1a). In fact, 69.9% of repeated elevations and 35.1% of one-time elevations were in obese women (with BMI >30 kg/m^2^), although they represented only 14.6% of the study sample.

**Table 2:** Multivariable-Adjusted Odds Ratio for C-Reactive Protein Elevations Above 10 mg/L.†

Characteristics		OR for one-time elevation above10 mg/L†	OR for repeated elevation above10 mg/L†
		(95% CI)	(95% CI)
Age (per year)		1.02 (0.98,1.07)	0.99 (0.93,1.05)
Gender (ref: female)			
	male	0.47 (0.32,0.71)^***^	0.29 (0.12,0.70)^**^
Race (ref: Caucasian)			
	African-American	1.33 (0.96,1.84)	1.46 (0.86,2.47)
Income(ref: <25 K/year)			
	25–50 K/year	0.97 (0.68,1.37)	0.57 (0.35,0.96)*
	50–75 K/year	0.83 (0.52,1.34)	0.43 (0.19,0.97)*
	>75 K/year	0.72 (0.40,1.29)	0.11 (0.01,0.89)*
Education(ref: high school)			
	college	0.89 (0.63,1.26)	1.02 (0.61,1.71)
	graduate school	1.22 (0.75,2.01)	0.90 (0.36,2.25)
Smoking status (ref: never smoker)			
	current smoker	1.49 (1.03,2.17)*	1.26 (0.72,2.23)
	former smoker	1.56 (1.05,2.32)*	0.88 (0.44,1.75)
Physical activity (per 100 EU)		0.98 (0.92,1.04)	0.93 (0.83,1.04)
Alcohol(ref: non-drinker)			
	<1 glass a day	0.89 (0.62,1.30)	0.78 (0.43,1.42)
	>1 glass a day	1.12 (0.77,1.65)	0.98 (0.52,1.87)
BMI (per kg/m^2^)‡		1.14 (1.09,1.18)^***^	1.35 (1.25,1.45)^***^
BMI squared‡		0.998 (0.996,0.999)*	0.993 (0.990,0.996)^***^
Male*BMI (per kg/m^2^)‡ ^||^		0.94 (0.87,1.02)	0.76 (0.65,0.89)^***^
Male*BMI-squared‡ ^||^		1.008 (1.002,1.013)^**^	1.017 (1.009,1.026)^***^
Comorbidities			
	hypertension	0.96 (0.58,1.59)	1.69 (0.91,3.14)
	diabetes mellitus	1.07 (0.54,2.13)	0.57 (0.20,1.57)
	hyperlipidemia	0.93 (0.59,1.48)	1.15 (0.58,2.26)
	respiratory conditions	1.27 (0.83,1.95)	1.39 (0.75,2.56)
	cancer	1.17 (0.46,2.97)	§
Medication use			
	sex hormones	1.90 (1.26,2.86)^**^	2.43 (1.34,4.43)^**^
	aspirin	0.85 (0.37,1.95)	0.48 (0.11,2.22)
	anti-hypertensives	0.46 (0.12,1.71)	0.66 (0.19,2.27)

Abbreviations; K, 1,000: BMI, body-mass index: OR, odds ratio: CI, confidence intervals: EU, exercise units:

* , **, *** denotes significance at 5%, 1%, 0.1% level, respectively.

† Multinomial logistic regression was used to calculate adjusted odds ratio, which is the ratio of odds of having either one-time or repeated CRP elevation (>10 mg/L) relative to the odds of having no CRP elevation (reference group). All predictors were measured at the initial CRP evaluation visit.

‡ BMI is centered at the overall sample mean (26.7).

§ There is no observation with history of cancer and repeated CRP elevation.

|| Male*BMI denotes the interaction between male gender and BMI; male*BMI-squared is the interaction between male gender and the square of BMI.

In the 328 participants who had at least one CRP elevation above 10 mg/L, the proportion with a one-time elevation was only 68.6%; nearly a third of those with CRP elevations above 10 mg/L had repeated elevations. Female gender, low income, hypertension, and high BMI were associated positively with the odds of CRP elevation being repeated, although the magnitude of the association with BMI was smaller in men than in women (Table 3 and Figure 1b). The most influential predictors in the model were BMI and gender: Their generalized Spearman correlation coefficients with the model-predicted log odds (the linear combination in the right hand side of the model) were 0.112 for BMI and 0.34 for gender; all other predictors had generalized Spearman correlations (with the model-predicted log odds) that were no greater than 0.24.

**Table 3:** Multivariable-Adjusted Odds Ratio for Repeated CRP Elevation Above 10 mg/L Among Those With at Least one CRP Elevation Above 10 mg/L*.

Characteristics		OR (95%CI)	*P* value
Male		0.21 (0.07,0.57)	.002
Income (ref: <25 K/year)			
	25 K-50 K/year	0.58 (0.32,1.03)	0.06
	50 K - 75 K/year	0.45 (0.18,1.10)	0.08
	> 75 K/year	0.09 (0.01,0.80)	0.03
Hypertension		2.26 (1.09,4.65)	0.03
BMI (kg/m^2^)†		1.13 (1.07,1.19)	<.001
Male*BMI† ‡		0.904 (0.819,0.997)	0.04
BMI squared†		0.994 (0.990,0.998)	0.002
Male*BMI-squared† ‡		1.010 (1.002,1.018)	0.02

Abbreviations; K, 1,000: BMI, body-mass index: OR, odds ratio: CI, confidence intervals:

* Multiple logistic regression was used to calculate the adjusted odds ratios.

† BMI is centered at the mean (32.4) of those with at least one CRP elevation.

‡ Male*BMI denotes the interaction between male gender and BMI; male*BMI-squared is the interaction between male gender and the square of BMI.

**Figure 2:**
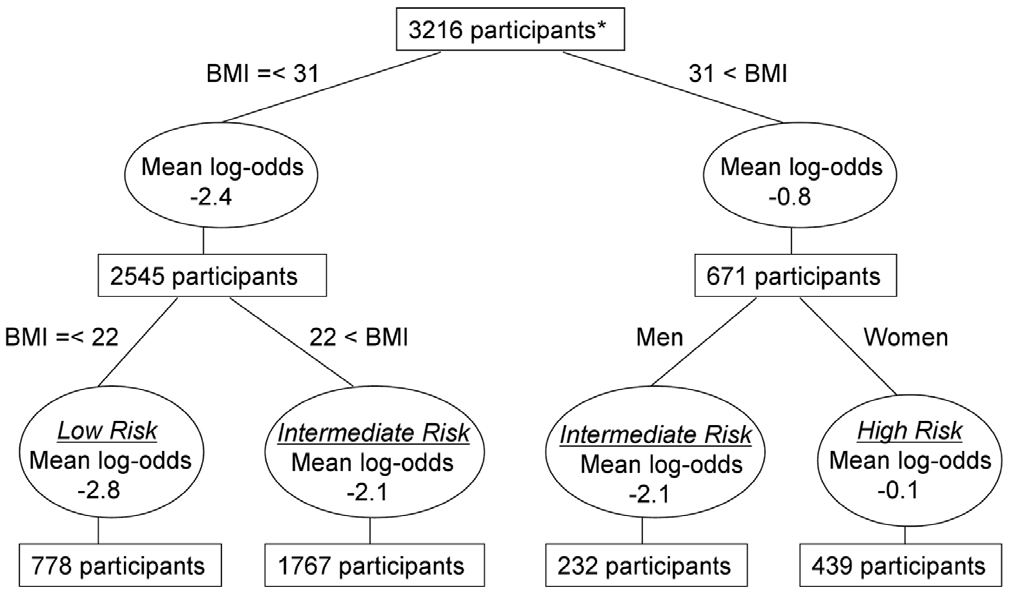
Risk stratification by baseline body mass index (BMI) and gender. Regression tree analysis was performed for the log odds of a CRP elevation being repeated (as predicted by the logistic regression model) with BMI and gender as predictors.

**Figure 3:**
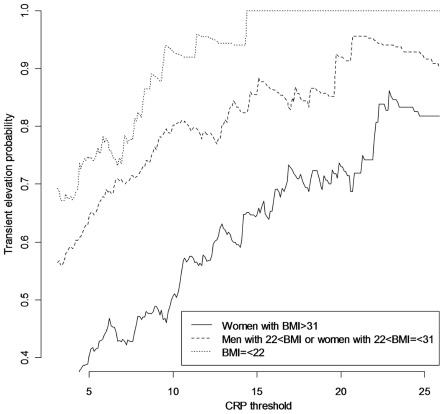
Probability of above-threshold CRP elevations being transient (one-time elevations), as function of CRP threshold. Probabilities in strata defined by gender and body mass index (BMI) were shown.

Regression tree analysis was then used to identify subgroups with distinct risks for a CRP elevation being repeated, with the model-predicted log odds as the outcome and BMI and gender as predictors. (Figure 2) Three groups were identified: women with BMI >31 kg/m^2^ (highest risk), men with BMI >22 kg/m^2^ and women with BMI above 22 kg/m^2^ but no more than 31 kg/m^2^ (intermediate risk), and men and women with BMI < = 22 kg/m^2^ (lowest risk). The probability that a CRP elevation is a one-time elevation was lowest, at 51%, in the highest risk group (women with BMI >31 kg/m^2^) compared to 82% in the rest of the cohort. Figure 3 depicts the probability of a CRP elevation being a one-time elevation (transient elevation probability) as a function of CRP threshold in the 3 risk groups defined by BMI and gender. For these “BMI-gender” defined groups, as Table 4 illustrates, there is considerable variability in the specific CRP thresholds needed to achieve a given level of transient elevation probability. In the intermediate-risk group, the probability that a CRP elevation is a one-time elevation is 80% at the usual threshold of 10 mg/L, but in women with BMI >31 kg/m^2^, the probability that a CRP elevation is a one-time elevation was 80% only at the much higher threshold of 22 mg/L.

**Table 4:** CRP Thresholds (in mg/L) Required to Achieve Varying Probabilities of Above-Threshold Elevations Being One-Time (Transient Elevation Probabilities) in Sub-Groups Defined by BMI and Gender.

Desired transient elevation probability	CRP threshold needed in low-risk group (BMI ≤22 kg/m2)	CRP threshold needed in intermediate-risk group (Men with BMI >22 and women with BMI >22 and ≤31 kg/m2)	CRP threshold needed in high-risk group (Women with BMI >31 kg/m2)
70%	4.4	6.5	16.8
75%	5.4	8.8	21.3
80%	7.7	10.0	22.1
85%	8.3	14.6	22.9
90%	9.4	19.7	27.8
95%	11.4	20.7	42.7

## Discussion

In the CARDIA study, the prevalence of elevated CRP values over 10 mg/L was 9.9%, with approximately 3% of the sample showing repeated elevations over 10 mg/L over 13 years. Importantly, these prevalences were generated *after* excluding those who reported being sick in the 24 hours prior to measurement. Female gender, low income, high BMI, and the use of sex hormones were independently associated with increased odds of repeatedly elevated CRP, relative to no CRP elevation above 10 mg/L. Previous studies of predictors of very high (>10 mg/L) CRP have not separated one-time CRP elevation from repeated elevation, but identified the same risk factors [5]–[7], [24], [25]. Consistent with previous studies, we also found that the relationship between BMI and the odds of very high CRP (both one-time and repeated) was not linear and was modified by gender [5], [7], [14], [15]. At higher levels of BMI in women, it appears that repeated CRP elevation is almost normative.

The major new finding from this study is that female gender, low income, high BMI and doctor-diagnosed hypertension, all independently increased the odds of a CRP elevation being repeated rather than one-time, with the association with BMI being non-linear and different by gender. All these characteristics tend to persist over time, suggesting that repeated CRP elevation represent a result of chronic processes [26]–[29].

Low income and female gender are both associated with greater levels of perceived stress [30], and chronic stress is a risk factor for inflammation [31]. Hypertension may also serve as a marker for chronic stress [32], as does obesity [33]. In addition, adipose tissue is a known source of CRP in humans [34]. Chronic conditions such as osteoarthritis, even if subclinical, are more likely in obese persons and can lead to elevated CRP, apart from inflammation caused directly by adipose tissue metabolism.

There are several possible explanations for our finding of gender differences in the relationship between high BMI and repeated CRP elevation. Higher estrogen levels may partly account for higher CRP levels in women, since this and previous studies have shown that exogenous estrogen therapy increases CRP levels [35], [36]. Estrogen levels are also positively associated with BMI in women [37]. Obesity is associated with greater susceptibility to infections [38], and therefore obesity-associated increase in frequency of infection may contribute to obesity-associated recurring increases in CRP. Whether there is any gender difference in obesity-associated susceptibility to infection warrants further research. Leptin, a hormone produced by adipose tissue, is higher in women compared with men, which may mediate some of the sex differences in the obesity-inflammation association [14], [39]. Gender differences in fat distribution, such as the greater accumulation of subcutaneous fat in women than in men which partially accounts for gender differences in CRP levels [40], may also play a role.

This study also demonstrated that the probability of a CRP measurement above 10 mg/L being one-time (transient) elevation varied significantly across subgroups, with the transient elevation probability being lowest (only 51%) in obese women (with BMI >31 kg/m^2^). To get transient elevation probabilities of 80%, the CRP threshold should be 22 mg/L in obese women.

Our study has some note-worthy limitations. First, CRP measurements were excluded in those who reported having been sick during the 24 hours preceding their blood draw; but since CRP levels stay elevated for 7 to 12 days after an acute inflammatory condition [41], the 24-hour look-back period may not be sufficient. However, under 2% of the analytic sample had acute inflammation (with one CRP measurement above 10 mg/L and others 3 mg/L or lower), suggesting that the 24-hour retrospective report excluded most individuals with acute inflammation. Second, we did not account for the temporal ordering of CRP measurements in our analyses, which leaves open the possibility that an isolated CRP elevation in year 20 represents new-onset chronic inflammation, not transient acute CRP elevation. But, the prevalence of CRP elevation above 10 mg/L did not increase with age (see table 1 and 2) or time/year. In addition, among those with CRP >10 mg/L in year 20, the proportion with repeated elevation was comparable to that in years 7 and 15 (data not shown), suggesting that we are not underestimating persistence of CRP elevation in year 20. Third, repeatedly elevated measurements of CRP could, in some participants, represent recurring acute inflammation rather than chronic inflammation; we could not distinguish between the two from three CRP measurements spread out over 13 years, and discounted the possibility of recurring acute inflammation. It should be noted however, that the risk factors identified here, namely, female gender, low income, high BMI and doctor-diagnosed hypertension may be associated with recurring acute inflammation rather than chronic inflammation – a question that requires considerably more detailed longitudinal data than are currently available through CARDIA. Lastly technical error in CRP measurement was fairly large in the study; while this could have led to some spurious elevations above 10 mg/L, it is unlikely that a person whose true value was several mg/L lower than 10 would have an observed value >10 mg/L.

Despite these limitations, our results have important implications for both clinical uses of CRP and epidemiological and clinical research studies on CRP. We have made several novel findings. We demonstrated that a significant proportion of people who do not report being sick (in the last 24 hours) have levels of CRP over 10 mg/L, which persist (or are at least, recurring) over 13 years. While our results are confirmatory that female gender, high BMI, sex hormone use and low income are chronic factors that are associated with CRP over 10 mg/L at a single cross-sectional timepoint, we also find that these chronic factors contribute to persistent elevated levels of CRP. These findings suggest that the current convention in research studies of excluding anyone with CRP >10 mg/L likely excludes many with chronic inflammation who are at the highest risk for poor health outcomes, and likely introduces bias into study findings.

We propose that epidemiologic studies that rely on a single measurement of CRP should not automatically discount all measurements above 10 mg/L as indicators of acute inflammation. Ideally, CRP should be measured repeatedly. If repeated measurements are not feasible and only a one-time measurement of CRP is available, participant-specific thresholds may be called for. In particular, a CRP threshold higher than 10 mg/L is needed to distinguish acute from chronic inflammation in obese women.
