# Correction: Gender, Obesity and Repeated Elevation of C-Reactive Protein: Data from the CARDIA Cohort

**DOI:** 10.1371/annotation/5459a88b-e488-4d4c-bd59-e7e8e99c8867

**Published:** 2012-07-12

**Authors:** Shinya Ishii, Arun S. Karlamangla, Marcos Bote, Michael R. Irwin, David R. Jacobs, Hyong Jin Cho, Teresa E. Seeman

The Funding section was missing information. The complete Funding statement reads: "This work was supported by the CARDIA contracts (N01-HC-48047 – N01-HC-48050 and N01-HC-95095), by grants P30 AG028748, 5R01 AG26105-3 to A.K., R01-AG034588 and R01-AG026364 to M.R.I., and P30 AG017265 to T.E.S. from the National Institute on Aging of the US National Institutes of Health, and VA Greater Los Angeles Healthcare System Geriatric Research, Education and Clinical Center; and VA Advanced Geriatrics Fellowship to S.I. The funders had no role in study design, data collection and analysis, decision to publish, or preparation of the manuscript."

The legend for Figure 1 was incorrect. The correct legend for Figure 1 reads: The predicted probabilities were computed from multiple logistic regression models while all other covariates were held constant (at reference values for categorical covariates and median values for continuous covariates). Panel A. Model-predicted probabilities of one-time and repeated CRP elevation. Panel B. Model-predicted probabilities of repeated CRP elevation among those with one or more CRP elevations above 10mg/L. 

